# The complete mitochondrial genome sequences of two insect-tea producers in Pyralidae (Lepidoptera) from South China: *Pyralis farinalis* and *Orthopygia glaucinalis*

**DOI:** 10.1080/23802359.2019.1687030

**Published:** 2019-11-06

**Authors:** Xin Mao, Hao-Xi Li, Mao-Fa Yang

**Affiliations:** a College of Forestry, Guizhou University, Guiyang, PR China;; b Institute of Entomology, Guizhou Provincial Key Laboratory for Agricultural Pest Management of the Mountainous Region, Guizhou University, Guiyang, PR China;; c College of Tobacco Sciences, Guizhou University, Guiyang, PR China

**Keywords:** Mitogenome, *Pyralis farinalis*, *Orthopygia glaucinalis*, Pyralidae

## Abstract

Complete mitochondrial genomes of *Pyralis farinalis* and *Orthopygia glaucinalis* were sequenced, respectively. Both contains 13 protein-coding genes (PCGs), 22 tRNA, two rRNA genes, and one AT-rich region. *Pyralis farinlis* mitogenome was 15,204 bp, with 11,234 bp coding 3732 aa. The rRNA had 1004 bp LSU and 802 bp SSU. Mitogenome of *O. glaucinalis* was 15,032 bp, with 11,038 bp coding 3668 aa. The rRNA contained 1406 bp LSU and 814 bp SSU. All PCGs used TAN as stop codon, except for both ND4 and ND5 of *O. glaucinalis*. Phylogenetic relationship of both species was also shown with 13 references.

Insect tea is a traditional health beverage in China. They were made by faeces obtained from caterpillars after feeding on special plant leaves (Bai 2010). So far, a total of seven species in Lepidoptera were documented as insect-tea producers (Shang et al. 2011; Liu et al. 2013). Among them, *Pyralis farinalis* and *Orthopygia glaucinalis* were popular in South China.

In August 2012, the samples of *P. farinalis* were collected in tea garden of Chishui County, Guizhou Province, China (E 105°12′00″, N 28°10′9″) while *O. glaucinalis* were gathered in Congjiang County, Guizhou Province, China (E 108°15′14″, N 25°13′2″). Then all the insects were fed in the Institute of Entomology, Guizhou University, Guiyang, China (E 108°16′14″, N 25°13′2″). Both the voucher of *P. farinalis* and *O. glaucinalis* with their genome DNA were deposited in the Institute of Entomology, Guizhou University, Guiyang, China (GUGC), accession number of them were GUGC-IPP-01801, GUGC-IPP-01901, respectively. Total DNA was extracted from the entire body of larva with Qiagen DNeasy kit (Venlo, the Netherlands) as the manufacture instruction. Raw data were outputted on an Illumina Novaseq 6000 platform at Berry Genomics (Beijing, China) for 150 bp paired-end reads. The reads were assembled* de novo* to draft mitogenome with NOVOPlasty version 2.7.2 (Dierckxsens et al. 2017). COI sequence of Pyralidae sp. (MK559413) was used as seed sequence for assembly. Then, Genes were annotated on mitogenome with Mitoz function in Mitoz version 2.4 (Meng et al. 2019). The results exhibited that 37 typical invertebrate mitochondrial genes (13 PCGs, 22 tRNAs, and 2 rRNAs) and the AT-rich region (D-loop) were identified from both mitochondrial genomes. Annotated sequences were submitted to Genbank with accession number MN442120 for *P. farinalis* and MN461479 for *O. glaucinalis*.

The circular mitogenome of *P. farinalis* was 15,204 bp in size, including 13 protein-coding genes (PCGs), 22 transfer RNAs (tRNAs), and 2 ribosomal RNAs (rRNAs). The nucleotide composition of the *P. farinalis* was obviously biased towards A + T (78.4%). There was 11,234 bp for the total length of 13 PCGs, which was used for encoding 3732 amino acids. The most common initiation codon was ATN within 13 PCGs. All PCGs of both mitogenome used TAN as stop codon, except for ND4 using TA. The length of LSU and SSU of rRNA gene were 1004 and 802 bp, respectively.

The mitogenome of *O. glaucinalis* was a circular molecule of 15,032 bp in length that contained 37 genes (13 PCGs, 22 tRNAs, and 2 rRNAs). In the total mitochondrial genome, the A + T content accounted for 79.2%. The total length of 13 PCGs was 11038 bp, encoding 3668 amino acids. ATN was discovered as initiation codon among all PCGs. Eleven PCGs of both mitogenomes used TAN as stop codon. However, ND4 and ND5 had the incomplete termination codon TA. The LSU (16S) of rRNA gene was 1406 bp and SSU (12S) was 814 bp.

For constructing phylogenetic tree, we used nucleotides sequences of 13 PCGs from 13 reference sequences. Among them, 12 were from other species within families Crambidae and Pyralidae. *Drosophila melanogaster* from Diptera was used as outgroup. Neighbour-joining tree (Saitou and Nei 1987)was built with Tamura–Nei Model and cost matrix as by Geneious Prime 2019.1.3 (Biomatters Ltd., Auckland, New Zealand). Results characterized that both species were clustered with family Pyralidae, along with other nine species. Species from family Crambidae were clustered separately (Figure 1). As a result, mitogenome data could provide more information on identification of insect tea producers.

**Figure 1.**
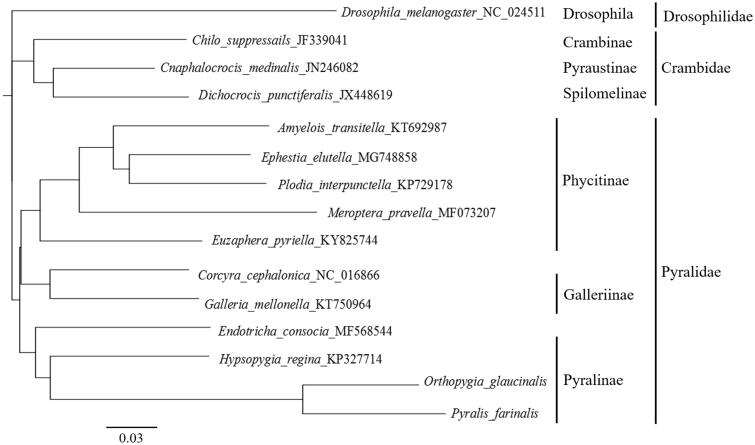
Neighbour-Joining tree was built under the Tamura–Nei Model and cost matrix as by Geneious Prime 2019.1.3.
